# Transfer Entropy Estimation and Directional Coupling Change Detection in Biomedical Time Series

**DOI:** 10.1186/1475-925X-11-19

**Published:** 2012-04-13

**Authors:** Joon Lee, Shamim Nemati, Ikaro Silva, Bradley A Edwards, James P Butler, Atul Malhotra

**Affiliations:** 1 Harvard-MIT Division of Health Sciences and Technology, Cambridge, MA, USA; 2 Massachusetts Institute of Technology, Cambridge, MA, USA; 3 Harvard Medical School, Division of Sleep Medicine, Brigham and Women's Hospital, Boston, MA, USA

## Abstract

**Background:**

The detection of change in magnitude of directional coupling between two non-linear time series is a common subject of interest in the biomedical domain, including studies involving the respiratory chemoreflex system. Although transfer entropy is a useful tool in this avenue, no study to date has investigated how different transfer entropy estimation methods perform in typical biomedical applications featuring small sample size and presence of outliers.

**Methods:**

With respect to detection of increased coupling strength, we compared three transfer entropy estimation techniques using both simulated time series and respiratory recordings from lambs. The following estimation methods were analyzed: fixed-binning with ranking, kernel density estimation (KDE), and the Darbellay-Vajda (D-V) adaptive partitioning algorithm extended to three dimensions. In the simulated experiment, sample size was varied from 50 to 200, while coupling strength was increased. In order to introduce outliers, the heavy-tailed Laplace distribution was utilized. In the lamb experiment, the objective was to detect increased respiratory-related chemosensitivity to *O*_2 _and *CO*_2 _induced by a drug, domperidone. Specifically, the separate influence of end-tidal *PO*_2 _and *PCO*_2 _on minute ventilation $\left({\dot{V}}_{E}\right)$ before and after administration of domperidone was analyzed.

**Results:**

In the simulation, KDE detected increased coupling strength at the lowest SNR among the three methods. In the lamb experiment, D-V partitioning resulted in the statistically strongest increase in transfer entropy post-domperidone for $PO_{2}\to {\dot{V}}_{E}$. In addition, D-V partitioning was the only method that could detect an increase in transfer entropy for $PCO_{2}\to {\dot{V}}_{E}$, in agreement with experimental findings.

**Conclusions:**

Transfer entropy is capable of detecting directional coupling changes in non-linear biomedical time series analysis featuring a small number of observations and presence of outliers. The results of this study suggest that fixed-binning, even with ranking, is too primitive, and although there is no clear winner between KDE and D-V partitioning, the reader should note that KDE requires more computational time and extensive parameter selection than D-V partitioning. We hope this study provides a guideline for selection of an appropriate transfer entropy estimation method.

## Introduction

In multi-variable time series analysis, a common subject of interest is the coupling among the variables. One promising measure of the coupling strength between two time series is transfer entropy [1,2], which quantifies the amount of information transfer from one variable to the other. Importantly, transfer entropy is non-parametric and can capture non-linear coupling effects. This property can be useful in analyzing complex systems where interactions among sub-systems are expected to be non-linear and where minimal a priori knowledge is available. Furthermore, transfer entropy is an asymmetric measure that conveys directional information. Unlike mutual information, which can only quantify the amount of shared information between two variables, transfer entropy can elucidate directional relationships between variables. In fact, it has been shown that transfer entropy is a non-linear extension of Granger causality [3].

Given all these advantages, transfer entropy has been gaining popularity as a powerful analytic tool for characterizing complex physiologic networks. For instance, this new method has been suggested [4] and successfully applied to electroencephalograms and magnetoencephalograms to help elucidate complex neural phenomena such as consciousness under anesthesia [5], sensory functional integration [6], auditory neural assembly [7], attention related processing [8], and sensor-motor connectivity [9]. In the area of cardiovascular physiology, Katura and colleagues [10] have utilized the technique of transfer entropy to elucidate (or reject) causal relationships that explain sources of variability in the regulation of cerebral hemodynamics. In addition, a number of studies have employed variations of transfer entropy, such as cross-conditional entropy and conditional mutual information, to investigate relationships between cardiovascular variables as indicator of baroreflex function [11-14]. Conditional mutual information has been shown to be equivalent to transfer entropy [15].

In practice, one major challenge with transfer entropy estimation using biomedical signals is the estimation of Probability Density Functions (PDFs) from finite data with outliers. This estimation challenge is well discussed in [2,16]. Small sample size can stem not only from experimental and/or technical constraints but also from the need for stationarity; a non-stationary time series may have to be broken into quasi-stationary segments before analysis. This sample size issue can be a major hindrance for biomedical studies where data collection is often costly. Also, outliers frequently originate from either noise sources or underlying physiology.

Therefore, our objective in this study was to investigate several transfer entropy estimation methodologies in the context of biomedical signals with small sample size and with outliers. This study can be viewed as an extension of the study on mutual information estimation (two dimensions) by Khan and colleagues [17] to transfer entropy estimation (three dimensions). Furthermore, unlike most other transfer entropy applications, we focused on the ability to detect increases in coupling strength, rather than on detection of the existence of a significant coupling or on identification of time lags at which significant couplings exist. This is a common research topic of interest when one wishes to study how a given coupling varies in magnitude under different conditions while the existence of the coupling is known a priori (our application to respiratory physiology in this study is one example). To the best of our knowledge, no study has attempted to investigate the sensitivity of various transfer entropy estimation methods to changes in coupling strength in the biomedical domain.

Specifically, we studied three non-parametric techniques in this endeavor: fixed-bins, Kernel Density Estimation (KDE), and adaptive partitioning. The fixed-bin approach, in which a given time series is quantized by dividing the dynamic range into equally-spaced bins, is the simplest method that boasts the least computational complexity at the cost of inefficient bin allocation [16]. KDE has been shown to perform well in mutual information estimation using short time series [17] and hence was included in our study. For adaptive partitioning, we introduce a novel method that applies the Darbellay-Vajda (D-V) partitioning algorithm [18] to transfer entropy estimation. The D-V partitioning algorithm is equivalent to the adaptive partitioning introduced by Fraser and Swinney [2,19] and has been shown to be effective in mutual information estimation [20].

The comparison among the three transfer entropy estimation techniques was accomplished via both simulated and real-life signals. In both experiments, we analyzed the sensitivity of the techniques to increased non-linear coupling strength, under small sample size and presence of outliers. The purpose of the simulated experiment was to perform a rigorous comparison in a controlled setting. In order to address any coupling characteristics that were not represented by the simulated data, we also applied the transfer entropy estimation methods to measurements of spontaneous breathing respiratory flow, end-tidal carbon dioxide pressure (*PCO*_2_) and end tidal oxygen pressure (*PO*_2_), collected from lambs [21]. The objective of the lamb experiment was to detect changes in chemosensitivity, which is of major clinical interest. The method of transfer entropy provides a natural framework for quantification of directional influence that breath-by-breath changes in blood gases have on ventilation (via peripheral chemoreceptors located in the aortic arch and carotid bodies), while taking into account the delayed nature of the response (due to lung-to-chemoreceptors transport time, typically on the order of 2-4 breaths) and possible non-linearities [22]. Ventilatory control instabilities are important in a variety of pathological conditions, including Cheyne-Stokes breathing in congestive heart failure [23,24] and obstructive sleep apnea [25-27]. Although the specific mechanisms underlying each condition may vary, increases in the ventilatory sensitivity to hypoxia/hypercapnia (controller gain) have been shown to play a critical role in the pathogenesis of unstable breathing. An elevated hypoxic ventilatory response at high altitude is associated with the presence of unstable breathing [28]. Moreover, heightened dynamic hypercapnic responses delineate heart failure patients with periodic breathing (in the form of Cheyne-Stokes respiration) from those patients that exhibit stable breathing [29-31]. Additionally, in heart failure patients, elevated hypoxic and/or hypercapnic sensitivity [32] and the consequent presence of Cheyne-Stokes respiration itself [33] are important predictors of mortality.

## Methods

### Transfer Entropy Construction

Given two concurrently sampled time series *X *= {*x*_1_, *x*_2_, . . ., *x*_*N*_} and *Y *= {*y*_1_, *y*_2_, . . ., *y*_*N*_}, the transfer entropy from *X *to *Y*, termed *T*_*X*→*Y*_, can be derived from conditional entropies as follows:

(1) $$T_{X\to Y}=H\left(y_{i}|y_{i-t}^{\left(l\right)}\right)-H\left(y_{i}|y_{i-t}^{\left(l\right)},\;x_{i-\tau }^{\left(k\right)}\right)$$

(2) $$=\underset{y_{i},y_{i-t}^{\left(l\right)},x_{i-\tau }^{\left(k\right)}}{\sum }p\left(y_{i},y_{i-t}^{\left(l\right)},x_{i-\tau }^{\left(k\right)}\right)\text{log}\frac{p\left(y_{i}|y_{i-t}^{\left(l\right)},x_{i-\tau }^{\left(k\right)}\right)}{p\left(y_{i}|y_{i-t}^{\left(l\right)}\right)},$$

where *i *indicates a given point in time, *τ *and *t *are the time lags in *X *and *Y*, respectively, and *k *and *l *are the block lengths of past values in *X *and *Y*, respectively (see Appendix I for a complete derivation). The past values on which the conditional probabilities in (2) are conditioned are $x_{i-\tau }^{\left(k\right)}=\left\{x_{i-\tau -k+1},\;x_{i-\tau -k+2},\;\ldots ,\;x_{i-\tau }\right\}$ and $y_{i-t}^{\left(l\right)}=\left\{y_{i-t-l+1},\;y_{i-t-l+2},\;\ldots ,\;y_{i-t}\right\}$. In words, (1) implies that transfer entropy measures the reduction in uncertainty in *y*_*i *_given $x_{i-\tau }^{\left(k\right)}$ and $y_{i-t}^{\left(l\right)}$ in comparison with given only $y_{i-t}^{\left(l\right)}$. Note that *T*_*X*_→_*Y *_cannot be negative because $H\left(y_{i}|y_{i-t}^{\left(l\right)}\right)\geq H\left(y_{i}|y_{i-t}^{\left(l\right)},x_{i-\tau }^{\left(k\right)}\right)$ in (1); conditioning on another variable, $x_{i-\tau }^{\left(k\right)}$, cannot increase the uncertainty in *y*_*i*_. (Note that if base 2 logarithm is used, the unit of transfer entropy is bits, which has the interpretation of the amount of reduction in the average length of the optimal code needed to encode the target variable.)

Equation (2) is the most general definition of transfer entropy and provides maximum flexibility. However, the usual parameter choices in practice are *k *= 1 and *l *= 1 for computational reasons when sample size is small. In addition, here we set *t *= 1 under the assumption that the maximum auto-transfer of information occurs from the data point immediately before the target value in *Y*. For example, Francis et al. only considered lags of one breath in ventilation and *CO*_2 _for the same reason [30]. These choices of *k *= *l *= *t *= 1 are especially appropriate in biomedical experiments where time series length is usually short and the absolute values of auto-correlation functions tend to decrease monotonically as time lag increases. Also, the original definition in [1] implicitly assumed *t *= 1 by using a Markov chain; our choice can be seen as a first-order Markov process. Hence, we restrict the focus of this paper to these usual parameter choices by simplifying (2) to

(3) $$T_{X\to Y}\left(\tau \right)=\underset{y_{i},y_{i-1},x_{i-\tau }}{\sum }p\left(y_{i},\;y_{i-1},\;x_{i-\tau }\right)\text{log}\frac{p\left(y_{i}|y_{i-1},x_{i-\tau }\right)}{p\left(y_{i}|y_{i-1}\right)}$$

(4) $$=\underset{y_{i},y_{i-1},x_{i-\tau }}{\sum }p\left(y_{i},\;y_{i-1},\;x_{i-\tau }\right)\text{log}\frac{p\left(y_{i},y_{i-1},x_{i-\tau }\right)p\left(y_{i-1}\right)}{p\left(y_{i-1},x_{i-\tau }\right)p\left(y_{i},y_{i-1}\right)}.$$

Note that (3) and (4) explicitly show transfer entropy as a function of *τ*.

In practice, the foremost challenge in transfer entropy computation is estimating the joint probabilities in (4). The ensuing section describes the three different PDF estimation methodologies to be investigated in this study.

### Probability Density Function Estimation Methodologies

#### Fixed Bins

The simplest estimation approach to obtain the PDFs in (4) is to allocate data points to fixed, equally-spaced bins. In order to enhance robustness against outliers and sparse regions in the underlying distribution, we combined fixed binning with ordinal sampling (ranking). In ordinal sampling, the time series values in *X *and *Y *are substituted with their ranks in sorted *X *and *Y *, similar to most non-parametric statistical tests. The ranks are integers ranging from 1 (smallest value) to *N *(largest value). Let the transformed time series of *X *and *Y *be *U *= {*u*_1_, *u*_2_, . . ., *u*_*N*_} and *V *= {*v*_1_, *v*_2_, . . .,*v*_*N*_}, respectively (*U *and *V *contain integers that represent ranks).

Then, (4) can be rewritten in terms of *U *and *V *as follows:

(5) $$T_{U\to V}\left(\tau \right)=\underset{v_{i},v_{i-1},u_{i-\tau }}{\sum }p\left(v_{i},\;v_{i-1},\;u_{i-\tau }\right)\text{log}\frac{p\left(v_{i},v_{i-1},u_{i-\tau }\right)p\left(v_{i-1}\right)}{p\left(v_{i-1},u_{i-\tau }\right)p\left(v_{i},v_{i-1}\right)}.$$

Using the same number of bins, *Q*, in each dimension for simplicity, the fixed-bin technique estimates (5) as follows:

(6) $$T_{U\to V}\left(\tau \right)\approx \overset{Q}{\underset{a=1,b=1,c=1}{\sum }}\frac{m_{a,b,c}}{P}\text{log}\frac{m_{a,b,c}m_{b}}{m_{b,c}m_{a,b}},$$

where *a*, *b*, and *c *index bins along *v*_*i*_, *v*_*i*-1_, and *u*_*i*-*τ*_, respectively, and *P *is the total number of triplets of *v*_*i*_, *v*_*i*-1_, and *u*_*i*-*τ*_. Furthermore, *m*_*a*,*b*,*c*_, *m*_*a*,*b*_, and *m*_*b*,*c *_represent the number of data points in the intersection of the one-dimensional bins denoted by the subscript, whereas *m*_*b *_is the number of data points in the *b*th bin along the *v*_*i*-1 _dimension.

#### Kernel Density Estimation

In KDE, the PDF is estimated by summing individual distributions centered at each data point. The shape of the individual distributions is dictated by a chosen kernel, the magnitude of which decreases as the distance from the center of the distribution increases. Since distance is a crucial measure in KDE, ordinal sampling is not employed. In the three-dimensional space of *y*_*i*_, *y*_*i*-1_, and *x*_*i*-*τ*_, the joint probability at an arbitrary point, $\left({ỹ}_{i},{ỹ}_{i-1},{\tilde{x}}_{i-\tau }\right)$, can be estimated by normalizing the following:

(7) $$p({\tilde{y}}_{i},\;{\tilde{y}}_{i-1},\;{\tilde{x}}_{i-\tau })\approx \frac{1}{P}\sum_{j=1}^{P}\frac{1}{h_{y_{i}}h_{y_{i-1}}h_{x_{i-\tau }}}K\left(\frac{{\tilde{y}}_{i}-y_{i,j}}{h_{y_{i}}}\right)K\left(\frac{{\tilde{y}}_{i-1}-y_{i-1,j}}{h_{y_{i-1}}}\right)K\left(\frac{{\tilde{x}}_{i-\tau }-x_{i-\tau ,j}}{h_{x_{i-\tau }}}\right),$$

where *j *indexes the *P *data points and *h*_(·) _is the bandwidth for the dimension specified in the subscript. We utilized a scaled version of the rule of thumb specified in [34] to select the bandwidth:

(8) $$h_{\left(\cdot \right)}=1.06\alpha \hat{\sigma }P^{-1/5},$$

where *α *is a multiplier for scaling and $\hat{\sigma }$ is the sample standard deviation in the given dimension. In terms of *K*, several possible kernels exist but we employed the widely used Gaussian kernel in this study, which is defined as follows:

(9) $$K\left(u\right)=\frac{1}{\sqrt{2\pi }}e^{-0.5u^{2}}$$

The rest of the probabilities in (4) can be computed by marginalizing $p\left({ỹ}_{i},\;{ỹ}_{i-1},\;{\tilde{x}}_{i-\tau }\right)$.

#### Adaptive Partitioning using the Darbellay-Vajda Algorithm

We introduce an extension of the D-V algorithm to three-dimensional space for transfer entropy estimation. After ordinal sampling, the D-V algorithm recursively partitions the three-dimensional space defined by *v*_*i*_, *v*_*i*-1_, and *u*_*i*-*τ *_into cubes of varying sizes. Initially, the entire space is sliced into 8 equal cubes, where the boundaries are at the mid-points in the three dimensions. Using the 8 cubes, the following *χ*^2 ^statistic is computed to test the null hypothesis that data points are evenly distributed across the 8 cubes:

(10) $$s_{\chi^{2}}=\sum_{i=1}^{8}{\left(M_{i}-\mu_{M}\right)}^{2},$$

where *M*_1_, *M*_2_, . . ., *M*_8 _are the numbers of data points contained in the 8 cubes and *μ*_*M *_is the total number of data points divided by 8 (number of cubes). If $s_{\chi^{2}}>\chi_{95\%}^{2}$ (7) (at a 5% significance level and 7 degrees of freedom), then the null hypothesis is rejected and each of the 8 cubes is further partitioned into 8 smaller cubes in the same manner, and the recursion continues. If the null hypothesis is not rejected, the partitions in the current iteration are discarded and the current 8 cubes under scrutiny as a whole are taken as one partition. Cubes that contain no data point do not contribute to the transfer entropy estimation.

Appendix II shows a two-dimensional analogy of a partitioned space. It was generated based on 1,000 data points randomly sampled from a bivariate Gaussian distribution with covariance *σ*_*xy *_= 0.9 and unit variance, $\sigma_{x}^{2}=1$ and $\sigma_{y}^{2}=1$. Note that many squares with smaller sizes are allocated to densely populated parts of the space, whereas fewer partitions are created in sparse areas.

The result of the partitioning process is a finite number of cubes, *L*, each of which contains nonzero data points. By approximating each probability in (5) by counting, *T*_*U*→*V *_(*τ*) can now be estimated using the *L *partitions as follows:

(11) $$T_{U\to V}\left(\tau \right)\approx \overset{L}{\underset{k=1}{\sum }}\frac{n_{k}}{P}\text{log}\frac{n_{k}n_{k}^{v_{i-1}}}{n_{k}^{v_{i-1},u_{i-\tau }}n_{k}^{v_{i},v_{i-1}}},$$

where *P *is the total number of data triplets (*v*_*i*_, *v*_*i*-1_, and *u*_*i*-*τ*_), *n*_*k *_is the number of data points in the *k*th partition, and $n_{k}^{v_{i-1}}$, $n_{k}^{v_{i},v_{i-1}}$, and $n_{k}^{u_{i-\tau },v_{i-1}}$ are the numbers of data points in the entire data that are greater than or equal to lower bounds and less than upper bounds of the *k*th partition with respect to the dimensions in the superscript. For example, if the *k*th partition's lower and upper limits in *v*_*i*-1 _are 5 and 10, then $n_{k}^{v_{i-1}}$ is the total number of data points such that 5 ≤ *v*_*i*-1 _< 10, regardless of their *v*_*i *_and *u*_*i*-*τ *_coordinates.

Our transfer entropy estimation method with D-V partitioning has been implemented in MATLAB, and we made the code and an example data set (from the simulated experiment described in the next section) publicly available at PhysioNet [35] (http://physionet.org).

### Experiments

#### Simulation

The source time series, *X *= {*x*_1_, *x*_2_, . . ., *x*_*N*+2_} and the corresponding sink time series, *Y *= {*y*_1_, *y*_2_, . . ., *y*_*N*+2_}, were generated as follows:

$$\begin{array}{l} x_{i}=s_{x,i}+\nu_{x,i}\\ y_{i}=s_{y,i}+\nu_{y,i}, \end{array}$$

where the signal (*s*_*x*,*i *_and *s*_*y*,*i*_) and noise components (*ν*_*x*,*i *_and *ν*_*y*,*i*_) are

(12) $$\begin{array}{l} s_{x,i}\sim N\left(10,1\right)\\ s_{y,i}={\left(a\cdot s_{x,i-2}\right)}^{2}\\ \nu_{x,i}\sim L\left(0,b_{x}\right)\\ \nu_{y,i}\sim L\left(0,b_{y}\right), \end{array}$$

where ~ denotes sampling from a distribution, *a *is a coupling constant, and  and  represent the Gaussian (mean, variance) and Laplace (mean, scale) distributions, respectively. The Laplace distribution is an example of a heavy-tailed statistic and thus can better model the type of noise often encountered in physiological signals compared with Gaussian noise (e.g., occasional sighs and apneas in a ventilation time series or muscle artifacts in electrocardiography (ECG) recordings [36]). Equation (12) imposed a nonlinear relationship between *X *and *Y *(via the squaring) at a time lag of two. Sample size, *N*, was varied from 50 to 200 at a step of 50, representing the focus of this experiment on small sample size. Note that the lengths of *X *and *Y *were *N *+ 2 due to the lag of 2; *x*_*N*+1_, *x*_*N*+2_, *y*_1_, and *y*_2 _were discarded during transfer entropy estimation.

The coupling strength between *X *and *Y *was varied by increasing the signal-to-noise ratio (SNR) in both time series from 10 to 20 dB in steps of 1 dB. This increase in SNR was induced by increasing the coupling constant *a*. In order to maintain the same SNR in both *X *and *Y *, the noise power in *X *was gradually reduced by decreasing *b*_*x *_while holding the signal power constant, whereas the signal power in *Y *was gradually increased by increasing *a *accordingly while holding the noise power (i.e., *b*_*y*_) constant.

As the first step, we analyzed transfer entropy vs. time lag (*τ*, from 0 to 5 in steps of 1) with SNR = 20 dB and *N *= 200 using all three estimation methods. This analysis served not only as a comparison among the methods but also as a parameter selection step for the fixed-bin (*Q *was varied from 4 to 10 in steps of 2) and KDE (*α *was varied from 0.5 to 1.5 in steps of 0.25) methods.

With the selected values for the parameters *Q *and *α*, *X *and *Y *were randomly generated 100 times at each SNR level and the corresponding transfer entropies were computed using all three methods. Subsequently, one-sided Wilcoxon rank sum tests were conducted to judge whether there was a significant increase in transfer entropy at each step of increase in SNR with *p *< 0.05 considered to be statistically significant.

#### An Application to Respiratory Physiology

Using the three transfer entropy estimation methods, we quantified the impact of the administration of domperidone, a dopamine *D*_2_-receptor antagonist that increases carotid body sensitivity to *O*_2 _and *CO*_2 _[21]. We performed our analysis on recordings from 14 newborn lambs obtained at Monash University, Australia [21]. All surgical and experimental procedures conformed to the guidelines of the National Health and Medical Research Council of Australia and had the approval of the Standing Committee in Ethics in Animal Experimentation of Monash University.

**Figure 1:**
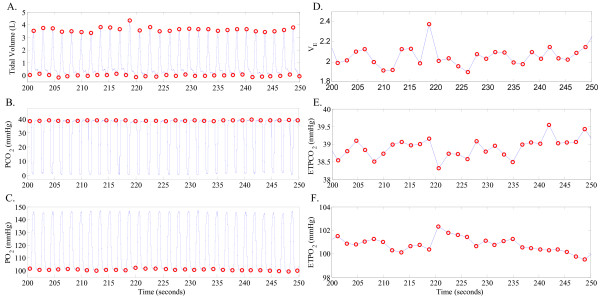
**Lamb experiment: example waveforms and derived time series**. Panels A, B, and C show examples of tidal volume, *PCO*_2 _and *PO*_2 _waveforms, respectively. The onset and offset of each inspiration are marked with an open circle in panel A. The ${\dot{V}}_{E}$ time series (in panel D) was derived from the tidal volume waveform as described in the text. The end-tidal *PCO*_2 _(*ET PCO*_2 _in panel E) and end-tidal *PO*_2 _(*ET PO*_2 _in panel F) time series were extracted from the measurements of *PCO*_2 _and *PO*_2 _in the end-expiratory air, respectively, as indicated by the open circles in panels B and C.

Measurements of spontaneous breathing respiratory flow, end-tidal *PCO*_2 _and end-tidal *PO*_2 _were obtained for a period of approximately 10 minutes before and following the intravenous administration of domperidone. Minute ventilation $\left({\dot{V}}_{E}\right)$ was calculated for each breath as *V*_*T*_/*T*_*tot*_, where *V*_*T *_is tidal volume and *T_tot _*is breath duration (see Figure 1 for example waveforms and derived time series). Breath-to-breath time series data of ${\dot{V}}_{E}$, end-tidal *PCO*_2 _and end-tidal *PO*_2 _were further band-pass filtered to remove any oscillations slower than (i.e., more breaths/cycle than) 20 breaths/cycle or faster than (i.e., fewer breaths/cycle than) 5 breaths/cycle (using a 7th-order Butterworth digital filter with band-pass frequency of 0.05-0.2 cycles/breath). The frequencies included in the resulting band-passed time series are consistent with the range of oscillations often observed during periodic breathing [21,25]. All extracted time series included at least ~ 200 data points. In a few cases where longer observations were available, the time series were divided into non-overlapping windows of size ~ 200 data points and the window with the largest variance was selected for further analysis. Our rationale behind this choice of data segment selection was to characterize the system at its most variable (or unstable) state during spontaneous breathing (see [37] for more information).

The three transfer entropy estimation methods were applied to the ${\dot{V}}_{E}$, *PO*_2_, and *PCO*_2 _time series from each lamb. Specifically, the following two directional information flows were investigated before and after the administration of domperidone: $PO_{2}\to {\dot{V}}_{E}$ and $PCO_{2}\to {\dot{V}}_{E}$. Unlike the simulation experiment, the time lag *τ *at which a significant coupling exists was unknown a priori. Hence, *τ *was varied between one and five breaths and the results for the time lag at which the value of transfer entropy was the largest and statistically significant were reported. Significant information flows were determined by Monte Carlo surrogates, i.e., temporally shuffled time series. In this experiment, transfer entropies were computed for 100 surrogates of the source time series (*PO*_2 _or *PCO*_2_), and the transfer entropy from the original source time series was deemed to be significant if it was greater than the 95th percentile of the surrogate results (as described in [38]). If no *τ *value resulted in a significant information flow, that particular time series pair was removed from further analysis. There was difficulty in selecting optimal *Q *and *α *values for the fixed-bin and KDE methods, respectively, due to the lack of a priori knowledge regarding coupling time lag. Therefore, arbitrary, but reasonable, choices of *Q *= 5 and *α *= 1 were made. Finally, increases in coupling strength caused by domperidone were tested using the Wilcoxon signed rank sum test.

## Results

### Simulated Experiment

**Figure 2:**
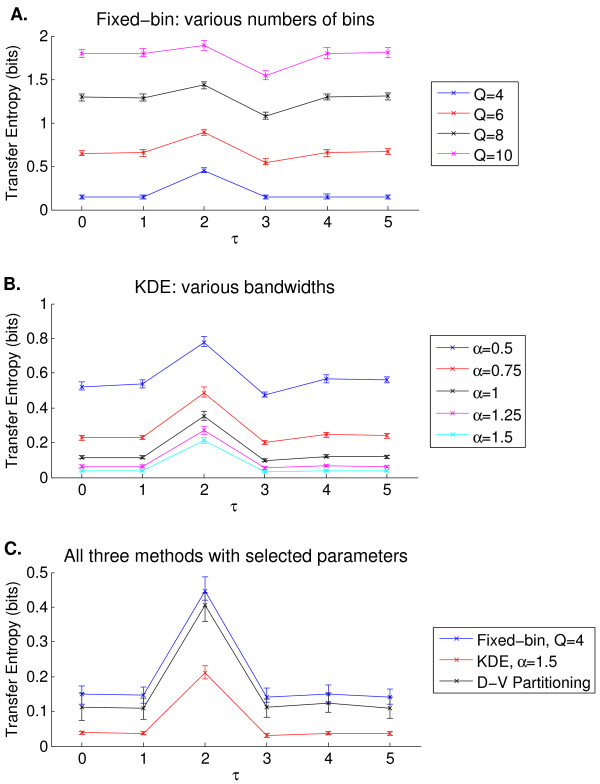
**Simulated experiment: parameter selection for the fixed-bin (panel A) and KDE (panel B) methods, and a comparison among the three transfer entropy estimation methods (panel C)**. Each panel shows a plot of median transfer entropy vs. time lag (*τ*), with SNR = 20 dB, *N *= 200, and 100 trials. Significant information transfer occurs only at *τ *= 2. The error bars represent interquartile range. Panels A and B show that sub-optimal parameters led to inflated transfer entropies at time lags of no coupling. Panel C compares the three methods after selecting *Q *= 4 and *α *= 1.5.

Figure 2 shows how the three transfer entropy estimation methods performed when *τ *was varied from 0 to 5 with *N *= 200 and SNR = 20 dB in the simulated experiment. Panels A and B illustrate transfer entropy estimation for different values of *Q *and *α *for the fixed-bin and KDE methods, respectively. Since one expects to see near zero transfer entropies at *τ *values other than 2, *Q *= 4 and *α *= 1.5 were selected for the rest of the experiment. Panel C of Figure 2 compares the three methods using the selected parameter values. All three methods allowed one to visually distinguish *τ *= 2 from other time lags. KDE was better at estimating low transfer entropies at *τ ≠ *2 at the cost of a reduced transfer entropy at *τ *= 2. Fixed-binning and D-V partitioning performed comparably, although D-V partitioning yielded lower transfer entropies at *τ ≠ *2.

**Figure 3:**
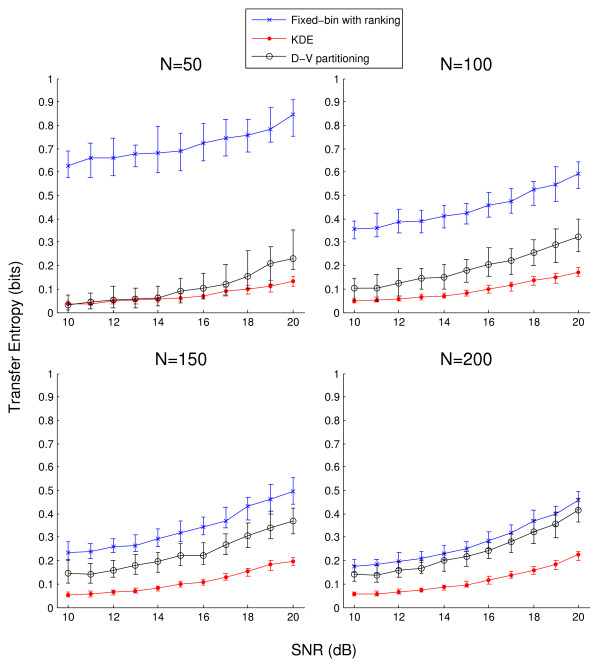
**Simulated experiment: transfer entropy as a function of SNR**. A comparison among the fixed-bin with ranking, KDE, and D-V partitioning methods. Based on 100 trials, each line shows median transfer entropy as a function of SNR, with four different sample sizes in separate panels. The error bars represent interquartile ranges. SNR represents both coupling strength and the contribution from outliers, and transfer entropy is expected to increase with increasing SNR. The corresponding one-sided rank sum test results are shown in Table 1.

The main results of the simulated experiment are shown in Figure 3 which illustrates the estimated transfer entropies from the 100 trials as a function of SNR. Following from Figure 2, *Q *= 4 and *α *= 1.5 for the fixed-bin and KDE methods, respectively. Overall, the fixed-bin method estimated higher transfer entropies at all lags than the other two methods, which was improved as *N *increased. KDE showed the smallest dynamic range in transfer entropy as SNR increased but was also associated with the smallest estimation variance at any given SNR. In fact, estimation variance with KDE was similar across different sample sizes, whereas the other two methods resulted in improved estimation variance as *N *increased.

Table 1 tabulates the one-sided rank sum test results of Figure 3 for detection of statistically significant increases in coupling strength. The two compared SNR levels are shown and statistically significant increases in transfer entropy were found to be sustained at and beyond the indicated SNR levels. In Table 1 all methods show the trend of detecting increased coupling at a lower SNR as *N *increases. Overall, KDE outperformed the other two methods in sensitivity to increasing coupling strength; for *N *≥ 100, KDE was able to distinguish 11 dB (*a *= 1.059) and 12 dB (*a *= 1.122). Although D-V partitioning was able to detect increases in transfer entropy at a lower SNR than the fixed-bin method when *N *= 50, the fixed-bin method slightly outperformed D-V partitioning at other *N *values.

**Table 1:** Simulated experiment: minimum SNR associated with sustained detection of statistically significant increases in transfer entropy

*N*	Fixed-bin	KDE	D-V partitioning
50	18 → 19 dB	15 → 16 dB	17 → 18 dB
100	15 → 16 dB	11 → 12 dB	17 → 18 dB
150	13 → 14 dB	11 → 12 dB	16 → 17 dB
200	11 → 12 dB	11 → 12 dB	13 → 14 dB

The minimum SNR at and beyond which statistically significant increases in transfer entropy are sustained according to the one-sided rank sum test. The two compared SNR levels are tabulated. See Figure 3 for visualization.

### Respiratory Physiology Experiment

**Figure 4:**
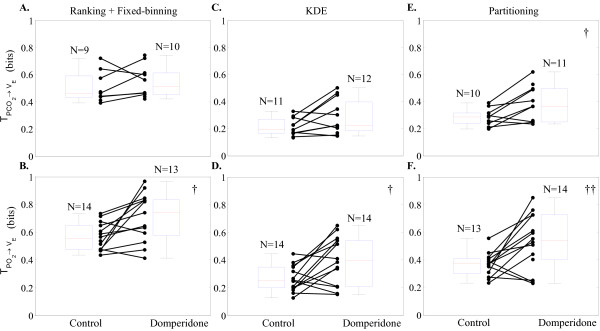
**Lamb experiment: comparison of the three transfer entropy estimation techniques**. Results from applying the fixed-bin, kernel density estimation, and adaptive partitioning techniques are presented in the first, second, and third columns, respectively. Within each panel, transfer of entropy during baseline (Control) and after drug administration (Domperidone) are compared. Each closed circle represents one animal, and the box-plots capture the group statistics (median and upper and lower quartiles). The control and post-domperidone transfer entropies from the same lamb are connected with a straight line; we expected to see as many lines with a positive slope as possible. The numbers above each box-plot indicate the total number of animals who passed the surrogate test of significance. † and †† indicate *p *< 0.05 and *p *< 0.01, respectively. Note that all three techniques indicate a statistically significant increase in *PO*_2 _chemosensitivity following domperidone administration; fixed-bin method in panel B: 0.55 bits [0.48 0.65] ([interquartile-range]) to 0.74 bits [0.58 0.83] (*p *< 0.05), kernel density estimation in panel D: 0.25 bits [0.20 0.35] to 0.40 bits [0.21 0.54] (*p *< 0.05), and adaptive partitioning in panel F: 0.37 bits [0.30 0.41] to 0.54 bits [0.40 0.73] (*p *< 0.01). Note that the partitioning technique indicated the largest increase in transfer entropy post-domperidone. Furthermore, only the partitioning technique revealed a significant increase in *PCO*_2 _chemosensitivity post-domperidone; panel E: 0.29 bits [0.24 0.32] to 0.36 bits [0.25 0.49] (*p *< 0.05).

Figure 4 illustrates how the estimated transfer entropy values changed for $PCO_{2}\to {\dot{V}}_{E}$ and $PO_{2}\to {\dot{V}}_{E}$ (first and second row, respectively), before ("Control") and after ("Domperidone") the administration of domperidone. The first, second, and third columns correspond to the fixed-bin, KDE, and D-V partitioning methods, respectively. Each closed circle represents one lamb, and the pre- and post-domperidone transfer entropies from the same lamb are connected with a straight line. Hence, we expect to see as many lines with a positive slope as possible. The number of subjects (*N*) varied among the different estimation methods as well as between pre- and post- domperidone since some cases did not pass the surrogate significance test. The group statistics are shown by the box plots.

Figure 4 shows that all three techniques were able to detect a statistically significant increase in transfer entropy for $PO_{2}\to {\dot{V}}_{E}$ (interpreted as increased chemosensitivity) post-domperidone. However, the D-V partitioning technique indicated the statistically strongest increase in transfer entropy following domperidone administration (*p *< 0.01). Moreover, only D-V partitioning detected a significant increase in the $PCO_{2}\to {\dot{V}}_{E}$ chemosensitivity post-domperidone (*p *< 0.05).

There are three other notable findings in Figure 4. First, fixed-binning led to higher transfer entropy values for both control and domperidone cases than the other two techniques. Second, unlike the simulation results, KDE did not show smaller estimation variance than the other two methods. Third, domperidone administration increased the range in transfer entropy in most panels in Figure 4.

## Discussion

In this paper, D-V partitioning was extended to three dimensions and, along with ranking, was used in the framework of transfer entropy estimation. Also, a comparison among three transfer entropy estimation techniques (namely traditional fixed-binning with ranking, state-of-the-art KDE, and adaptive partitioning) was conducted via a simulated experiment and an application to respiratory physiology. The focus of both studies was to investigate the methods' ability to detect increases in directional coupling strength under small sample size and outliers which are commonly encountered in biomedical studies. Although KDE detected increases in coupling strength at the lowest SNR level across different sample sizes in the simulation, D-V partitioning outperformed the other two methods in detecting increased chemosensitivity caused by domperidone in the lamb experiment. The respiratory chemoreflex system was a suitable example of non-linearity and non-stationarity. Several authors have reported evidence for non-linearity in the respiratory chemoreflex system [22,39]. Non-stationarity may also arise as a result of fluctuations in other variables/parameters such as cardiac output, respiratory rate, sleep-state, arousal-related changes in respiratory mechanics and the chemical control system, behavioral factors, or interventions. The application to respiratory physiology in this article demonstrated that the adaptive-partitioning-based transfer entropy estimation technique was more sensitive to increases in coupling strength for both the *PCO*_2 _and *PO*_2 _chemosensitivity than the other two methods. These results, which are based on non-invasive measurements of spontaneous breathing ventilatory variables, are in agreement with the experimental findings of Edwards and colleagues [21] based on the traditional hypercapnic and hypoxic responses (i.e., ratio of change in ventilation in response to an incremental increase in *PCO*_2 _or decrease in *PO*_2 _in the inspired air). Therefore, transfer entropy has the potential to be a useful surrogate marker of chemosensitivity, and may provide physiologists and physicians with a non-invasive tool to track patient response to pharmacological interventions (e.g., Acetazolamide).

Strictly speaking, transfer entropy measures the coupling strength of a causal link between two time series at a specific time lag and thus makes it difficult to attribute any observed change in transfer entropy to changes in chemosensitivity. For example, Porta et al. have also shown that cross-conditional entropy can be high with a relatively low level of baroreflex sensitivity [11]. Thus, any interchangeability in the current study between transfer entropy and chemosensitivity requires further investigation, since it is possible to increase transfer entropy by, for example, improving the SNR in the data acquisition system under the same level of chemosensitivity or changing the operating point of the feedback system in the direction of an improved SNR. The simulation corroborated the lamb experiment by enabling a controlled comparative analysis. For example, unlike the lamb experiment, the simulated experiment allowed us to control the amount of noise and forced the signals to be stationary throughout. Furthermore, the square function and Laplace noise simulated a non-linear coupling relationship and real-life noise, respectively, that are easy to interpret and control.

Both fixed-binning and D-V partitioning incorporated ordinal sampling, or ranking, which should have at least partially dealt with outliers and multi-modal PDFs. The only difference between the two methods is in how the three-dimensional space is partitioned, and here the efficient bin allocation of the D-V partitioning algorithm becomes a substantial advantage. Under non-uniform distributions that exhibit multiple peaks or varying concentrations of data points, fixed-binning is destined to function inefficiently by allocating fixed-size bins from the minimum to maximum observed values. On the other hand, the D-V partitioning adaptively adjusts bin size according to how evenly data are distributed in a given sub-region of the data space. The expected effectiveness of adaptive partitioning increases with greater deviation from a uniform distribution, and this advantage could partially explain the improved performance of the D-V partitioning method in the lamb experiment in comparison with the simulation.

Moreover, there are minimal adjustable parameters in the D-V partitioning algorithm (one can choose to change the significance level of the *χ*^2 ^test), whereas the fixed-bin and KDE methods require one to choose the number of bins and the bandwidth of the kernel a priori. In fact, KDE allows one more degree of freedom: selection of the kernel function, which was not analyzed in this study. Parameter selection can be challenging especially with real-life data where the true coupling characteristics (e.g., strength, direction, time lag, etc.) are often unknown. With the D-V partitioning technique, one can bypass the often arbitrary step of model parameter selection, and this can be a substantial advantage in practice.

The block lengths *k *and *l *as well as the time lag *t *were fixed at 1 in the current study similar to previous studies quoting computational reasons, despite the known limitation that transfer entropy is expected to be underestimated [1,38]. In cases where data size is sufficiently large and computational tractability is not a concern, one can attempt to optimize *k*, *l*, and *t*. For instance, Faes et al. discussed a greedy approach for conditional entropy that selects an optimal conditional pattern consisting of past data from two or more related signals [12]. Porta et al. studied the effects of conditional pattern length on cross-conditional entropy [40]. With respect to computational time, the three different methods are ranked in the following ascending order: fixed-bin, D-V partitioning, and KDE. Although KDE performed very well in the simulated experiment, the necessity to compute the distance between a given point and every point in the data set along each dimension makes it computationally intensive. However, it is worthwhile noting that computational time is not a major limiting factor in most research studies that only require retrospective analysis.

In a number of biomedical studies, small sample size and presence of outliers are often closely associated with physiological dynamics. A common focus of many research studies is to investigate how physiological systems behave under different conditions or respond to external interventions, naturally formulating a non-stationary problem. One way to analyze a non-stationary time series is to segment them into quasi-stationary intervals. This limits the number of data points in each segment and leads to the small sample size problem. This can be a major challenge to many parameter estimation algorithms in presence of noise, because they often require sufficiently long observations. In addition, physiological systems tend to exhibit non-linear behaviors which may cause the state of the system to abruptly change from its steady-state baseline, producing outliers. These outliers, originating from underlying physiological phenomena rather than noise, convey meaningful value and hence should not be removed for mere computational convenience. We remind the reader that our simulated experiment explicitly investigated the issues of small sample size and of outliers.

## Conclusions

In this article, we have shown that transfer entropy can detect changes in directional coupling between two biomedical time series. We have extended D-V partitioning to transfer entropy estimation and compared the performance of three transfer entropy estimation methods in detection of increased coupling strength: fixed-binning with ranking, KDE, and D-V partitioning with ranking. Based on the results of this study, fixed-binning, even with ranking, failed to clearly outperform the other two methods, while the comparison between KDE and D-V partitioning was inconclusive. However, D-V partitioning may be the most attractive option after taking into account computational time and the difficulty associated with parameter selection. We hope that this study provides a helpful guideline in selecting an appropriate transfer entropy estimation method.

## Appendix I

Given two concurrently sampled time series *X *= {*x*_1_, *x*_2_, . . ., *x*_*N*_} and *Y *= {*y*_1_, *y*_2_, . . ., *y*_*N*_}, the conditional entropy *H*(*x*_*i *_| *y*_*i*_) is defined as:

$$H\left(x_{i}|y_{i}\right)=\underset{x_{i},y_{i}}{\sum }p\left(x_{i},\;y_{i}\right)\text{log}\frac{p\left(y_{i}\right)}{p\left(x_{i},y_{i}\right)}.$$

Note that *H*(*x*_*i *_| *y*_*i*_) ≥ 0. Then, the transfer entropy from *X *to *Y*, termed *T*_*X*→*Y*_, can be derived from conditional entropies as follows:

(13) $$\begin{array}{llll} T_{X\to Y} & =H\left(y_{i}|y_{i-t}^{\left(l\right)}\right)-H\left(y_{i}|y_{i-t}^{\left(l\right)},\;x_{i-\tau }^{\left(k\right)}\right)\; & & \;\\ & =\underset{y_{i},y_{i-t}^{\left(l\right)}}{\sum }p\left(y_{i},\;y_{i-t}^{\left(l\right)}\right)\text{log}\frac{p\left(y_{i-t}^{\left(l\right)}\right)}{p\left(y_{i},y_{i-t}^{\left(l\right)}\right)}-\underset{y_{i},y_{i-t}^{\left(l\right)},x_{i-\tau }^{\left(k\right)}}{\sum }p\left(y_{i},\;y_{i-t}^{\left(l\right)},\;x_{i-\tau }^{\left(k\right)}\right)\text{log}\frac{p\left(x_{i-\tau }^{\left(k\right)},y_{i-t}^{\left(l\right)}\right)}{p\left(y_{i},y_{i-t}^{\left(l\right)},x_{i-\tau }^{\left(k\right)}\right)}.\; & & \;\\ & \; & \end{array}$$

But since $p\left(y_{i},\;y_{i-t}^{\left(l\right)}\right)=\sum_{x_{i-\tau }^{\left(k\right)}}p\left(y_{i},\;y_{i-t}^{\left(l\right)},\;x_{i-\tau }^{\left(k\right)}\right)$, the two arguments in the separate summations can be combined. Thus,

$$\begin{array}{c} T_{X\to Y}=\underset{y_{i},y_{i-t}^{\left(l\right)},x_{i-\tau }^{\left(k\right)}}{\sum }p\left(y_{i},\;y_{i-t}^{\left(l\right)},\;x_{i-\tau }^{\left(k\right)}\right)\text{log}\frac{p\left(y_{i-t}^{\left(l\right)}\right)p\left(y_{i},y_{i-t}^{\left(l\right)},x_{i-\tau }^{\left(k\right)}\right)}{p\left(y_{i},y_{i-t}^{\left(l\right)}\right)p\left(y_{i-t}^{\left(l\right)},x_{i-\tau }^{\left(k\right)}\right)}\\ =\underset{y_{i},y_{i-t}^{\left(l\right)},x_{i-\tau }^{\left(k\right)}}{\sum }p\left(y_{i},\;y_{i-t}^{\left(l\right)},\;x_{i-\tau }^{\left(k\right)}\right)\text{log}\frac{p\left(y_{i}|y_{i-t}^{\left(l\right)},x_{i-\tau }^{\left(k\right)}\right)}{p\left(y_{i}|y_{i-t}^{\left(l\right)}\right)}. \end{array}$$

## Appendix II

**Figure 5:**
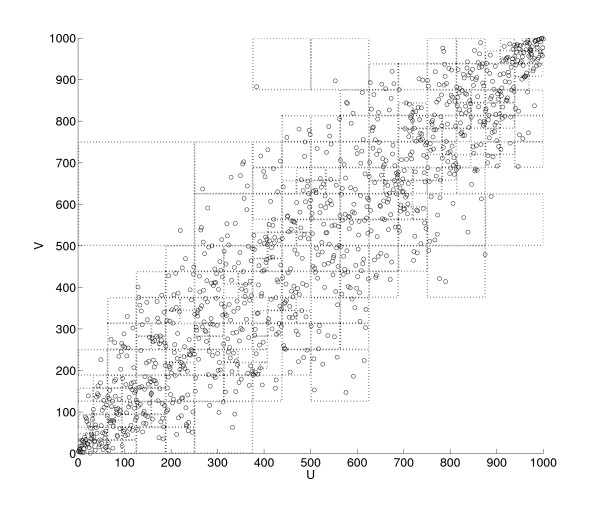
**A two-dimensional visualization of D-V partitioning**.

Figure 5 shows a two-dimensional visualization of D-V partitioning. The space contains 1,000 data points sampled from a bivariate Gaussian distribution with *σ_xy _*= 0.9, $\sigma_{x}^{2}=1$, and $\sigma_{y}^{2}=1$. This figure shows the transformed data space after ordinal sampling. The proposed transfer entopy estimation with the D-V partitioning algorithm extends this kind of partitioning to three dimensions.

## Competing interests

The authors declare that they have no competing interests.

## Authors' contributions

JL and SN conceived of the study, developed and implemented the D-V partitioning transfer entropy estimation algorithm, conducted the experiments, and wrote the entire manuscript. IS conceived of the study, contributed to the algorithm development, and wrote parts of the manuscript. BE supplied the lamb data and rigorously revised the manuscript. JB and AM thoroughly revised the manuscript.
